# Interaction between Autonomic Regulation, Adiposity Indexes and Metabolic Profile in Children and Adolescents with Overweight and Obesity

**DOI:** 10.3390/children8080686

**Published:** 2021-08-09

**Authors:** Valeria Calcaterra, Carlo Palombo, Mara Malacarne, Massimo Pagani, Giovanni Federico, Michaela Kozakova, Gianvincenzo Zuccotti, Daniela Lucini

**Affiliations:** 1 Pediatric Department, “V. Buzzi” Children’s Hospital, 20154 Milan, Italy; gianvincenzo.zuccotti@unimi.it; 2 Pediatric and Adolescent Unit, Department of Internal Medicine, University of Pavia, 27100 Pavia, Italy; 3 Department of Surgical, Medical, Molecular Pathology and Critical Area Medicine, University of Pisa, 56126 Pisa, Italy; carlo.palombo@unipi.it (C.P.); m.kozakova@int.med.unipi.it (M.K.); 4 BIOMETRA Department, University of Milan, 20133 Milan, Italy; mara.malacarne@unimi.it; 5 Exercise Medicine Unit, Istituto Auxologico Italiano, 20133 Milan, Italy; massimo.paganiz@gmail.com; 6 Pediatric Endocrinology and Diabetes Unit, Department of Clinical and Experimental Medicine, University of Pisa, 56126 Pisa, Italy; Giovanni.Federico@med.unipi.it; 7 Department of Biomedical and Clinical Science “L. Sacco”, University of Milan, 20157 Milan, Italy

**Keywords:** pediatric obesity, autonomic regulation, adiposity index, metabolic, children, adolescents, overweight, peripheral endothelium

## Abstract

Early obesity predicts initial modifications in cardiac and vascular autonomic regulation. The aim of this study was to assess the possible interaction between non-invasive measures of autonomic cardiovascular control and peripheral endothelium regulation in children with overweight and obesity. We involved 114 young subjects (77M/37F, 12.7 ± 2.2 years) with normal weight (NW, *n* = 46) to overweight or obesity (OB, *n* = 68). Multivariate statistical techniques utilizing a collection of modern indices of autonomic regulation, adiposity indexes and metabolic profile were employed. Resting values show substantial equivalence of data. Conversely, blood pressure variance is greater in NW/OB groups. The correlation matrix between major autonomic and metabolic/hemodynamic variables shows a clustered significant correlation between homogeneous indices. A significant correlation between metabolic indices and endothelial and autonomic control, mostly in its vascular end, was recorded. Particularly, the alpha index is significantly correlated with triglycerides (r = −0.261) and endothelial indices (RHI, r = 0.276). Children with obesity show a link between indices of autonomic and endothelial function, fat distribution and metabolic profile. The optimization of autonomic control, for instance by exercise/nutrition interventions, could potentially prevent/delay the occurrence of structural vascular damage leading to reduced cardiovascular health.

## 1. Introduction

Pediatric obesity remains an important health problem. The prevalence of obesity increased three times from 1975 to 2016 [1]; overweight or obesity were recorded in more than 40 million children aged under the age of 5 years and 340 million children and adolescents aged 5–19 years [2]. In Italy, 17% and 9.3% of school-aged children were overweight or obese [3].

Body-fat excess and distribution play critical roles for health risk [4]. Body mass index (BMI), waist circumference (WC) and/or waist-to-height ratio (WHtR) are the most widely used indices in clinical practice. Even though these parameters present serious limitations, particularly in children, because they do not accurately discriminate between lean and fat mass, they are considered good markers of metabolic and cardiovascular comorbidities [5,6]. Recently, new indexes of obesity, such as triponderal mass index (TMI) [7] and non-linear equations to estimate the fat mass [8], have been proposed as markers of cardiovascular risk. Additionally, ultrasonography may be used as a reliable method to measure subcutaneous and visceral fat for predicting health risk [9] related to obesity.

Obesity early in life predisposes individuals to later cardiovascular and metabolic alterations [10]. In particular, early obesity strongly predicts initial modifications in cardiac and vascular autonomic regulation [10]. Importantly, increased peripheral sympathetic activation and reduced cardiac vagal modulation, possibly leading to arterial hypertension, were reported in children with overweight and obesity [11]. The sympathetic activity might play a role in modulating the elastic properties of central [12,13] and peripheral arteries.

Although autonomic abnormalities are usually treated as an organic irreversible disorder, it was previously documented that autonomic abnormalities may be in a large part functional in children with overweight and obesity [12,13] and the optimization of body weight by following healthy diet and physical activity programs could be considered a crucial strategy in their prevention and treatment [14,15,16,17,18].

The aim of this study was to assess the possible interaction between non-invasive measures of autonomic cardiovascular control and peripheral endothelium regulation in children with overweight and obesity. To this end, we employed multivariate statistical techniques utilizing a collection of modern indices of autonomic regulation, adiposity indexes and metabolic profile [19,20].

## 2. Materials and Methods

### 2.1. Selection of the Patients

Given the exploratory nature of the investigation, we designed a retrospective study employing multivariate statistical techniques utilizing a collection of modern numerical and graphical tools considering the interplay between autonomic regulation, adiposity outline and metabolic profile [19,20].

We considered 114 young subjects (77M/37F, mean overall age: 12.7 ± 2.2 years) with normal weight (*n* = 46) to overweight or obesity (*n* = 68), consecutively referred as outpatients to the Pediatric Endocrinology and Diabetes Unit, Department of Clinical and Experimental Medicine, University of Pisa, Italy, for evaluation of metabolic status, between 1 February 2016 and 10 November 2016. Primary obesity, females and males, and a chronological age between 8 and 18 years were inclusion criteria. The presence of known secondary obesity conditions, use of any drugs, and concomitant chronic or acute illnesses were considered exclusion criteria.

In all patients, anthropometric data, metabolic evaluation, peripheral endothelium-dependent vasodilator capacity, ultrasound measurement of subcutaneous and visceral fat and autonomic evaluation were recorded.

### 2.2. Anthropometric Data

Height and weight were recorded in the morning, between 08:00 and 10:00 a.m. Standing height was measured using a Harpenden stadiometer (Holtain Ltd., Crosswell, UK) with a fixed vertical backboard and an adjustable head piece. The measurement was taken on the subject in an upright position, without shoes, with their heels together and toes apart, hands at sides, aligning the head in the Frankfort horizontal plane [21]. Weight was quantified with participants not wearing shoes and in light clothing, standing upright in the center of the scale platform (Seca, Hamburg, Germany) facing the recorder, hands at sides, and looking straight ahead [21]. Two measurements were taken for each parameter, and a third was obtained if a discrepancy was noted between the initial measurements for weight (>500 g) and height (>0.5 cm).

BMI as well as BMI z-scores were calculated using World Health Organization (WHO) reference values [22] to obtain worldwide valid results.

The study subjects were divided into two groups: normal weight group if BMI z-score was <1 and overweight/obese group if BMI z-score ≥1 [22].

In addition to BMI, BMI z-score and WC adiposity indexes, including WHtR, TMI, and a non-linear equation to estimate the fat mass were calculated as follows:

- WHtR = WC/Ht;
- TMI = weight (kg)/height (m)^3^ [23];
- Fat Mass = weight − exp (0.3073 × height^2^ − 10.0155 × weight^−1^ + 0.004571 × weight −0.9180 × ln(age) + 0.6488 × age^0.5^ + 0.04723 × male + 2.8055) [24] (exp = exponential function, ln = natural logarithmic transformation, male = 1, female = 0).

### 2.3. Metabolic Evaluation

Blood samples were collected by venipuncture, in the morning after overnight fasting [25].

As insulin resistance (IR) surrogates we considered the following:

HOMA-IR calculated as insulin resistance = (insulin × glucose)/22.5 [26];
TyG-index calculated as ln[fasting triglycerides (mg/dL) × fasting plasma glucose (mg/dL)/2] [27].

### 2.4. Endothelium-Dependent Vasodilation: RHI

Peripheral endothelium-dependent vasodilator capacity was estimated assessing reactive hyperemia index (RHI), a measure for endothelial function, by means of an EndoPAT 2000 device (Itamar Medical Ltd., Cesarea, Israel) as previously described [28,29]. RHI was calculated on peripheral artery tonometry (PAT) signal changes with a proprietary computerized algorithm. Finger probes were used to assess arterial pulse wave amplitudes at the fingertips.

Subjects, after overnight fasting, were in a supine position for at least 20 min before the test, in a quiet room, with comfortable lights and temperature. The Endo-PAT pneumatic probes were placed on both index fingers to register arterial pulse wave amplitudes, i.e., the difference between the highest and lowest points of a pulse wave. The subjects were requested to remain as still as possible during the whole measurement phase. Each recording comprised 5 min baseline testing, 5 min occlusion testing, and 5 min post-occlusion testing (hyperemic period).

Occlusion of the brachial artery was performed on the non-dominant upper arm by using a manometer cuff inflated to supra-systolic pressure (at least 40 mmHg above the systolic BP). The computerized algorithm automatically calculated the RHI; that is, the ratio of the mean pulse wave amplitude between 90 and 150 s after deflation divided by a pre-occlusion period value during 210 s before occlusion, divided by the same ratio for the control arm to correct for changes in systemic vascular tone.

### 2.5. Ultrasound

A standard ultrasound system (MyLab 70, Esaote, Genova, Italy) equipped with a 3.5 MHz phased-array probe was used to obtained cardiac images. Conventional B-mode, M-mode and Doppler echocardiography were used for the assessment of LV mass and other measures (not employed in the present study). Ultrasonography for evaluation of subcutaneous and visceral fat was performed in subjects placed in a supine position. Maximum subcutaneous abdominal fat thickness was measured 5 cm above the umbilicus, as the distance between skin-fat and fat-linea alba interfaces [30]. Visceral fat was measured one cm above the umbilicus as the distance between the internal face of retro-abdominal muscle and the anterior wall of the aorta [31].

### 2.6. Autonomic Evaluation

On the day of the study, all subjects arrived at the laboratory about 2 h after a light breakfast, avoiding caffeinated beverages and heavy physical exercise in the preceding 24 h. Recordings were always performed between 10:00 AM and 12:00 PM to account for circadian variations. After a preliminary 10-min rest period in a supine position, allowing for stabilization, arterial pressure (AP) waveforms, ECG, and respiratory activity were continuously recorded over a 10-min baseline and a subsequent 7-min period of active standing, and then stored on a PC.

As described previously [19,20], from the simultaneous autoregressive spectral analysis of the RR interval and SAP variability, a series of indices indirectly reflecting autonomic cardiovascular modulation are derived. The three major oscillatory components extracted from the continuous RR segment with an ad hoc SW tool are presented as absolute (in time) and normalized (dimensionless, pure numbers) units. The component around 0.25 Hz and strongly coherent with the major respiratory oscillation (*p* < 0.001) is defined as high frequency (HF), a lower frequency component (around 0.1 Hz) is called low frequency (LF), and the lowest frequency component (around 0.0–0.03 Hz) is called very low frequency (VLF, or DC) and is disregarded in short-term recordings. Additional methodological details have been recently published [19].

The simultaneous bivariate analysis of RR and systolic arterial pressure variability also permits extraction of the gain between systolic arterial pressure and coherent RR variability components [32]. The derived index (alpha) is strongly linked to RR variance and provides an approximate estimate of the SAP RR (open) loop. The combination of these indices is a strong predictor of cardiac events.

It is worth noting that in recent years, interest in new methods of analysis of heart rate variability (HRV), or the physiological underpinning of various derived indices, considered markers of the antagonistic sympathetic-parasympathetic neural balance of cardiac regulation, has increased substantially. Indeed, in the Medline database the entry “Heart Rate Variability” elicits more than 50,000 hits, suggesting that HRV is the de facto non-invasive standard for the assessment of cardiac autonomic regulation. There are continuing debates and intensive scrutiny into novel methods stimulated by the strong predictive capacity of HRV in cardiac and metabolic conditions, or on the possibility of guiding training and fostering results in athletes as well as in ambulatory subjects. In the last few years, with a data driven approach, we have tried to simplify understanding of the relationship between the multiple indices produced by computer analysis of cardiac and vascular beat-by-beat variability signals using a multivariate statistical approach. This method is based on various steps, in particular the use of dimension reduction tools, such as the exploratory factor analysis [20]. With this approach we intend to assess whether the transition from normal weight to overweight/obesity combines with a worsening in the overall autonomic nervous system (ANS) condition as captured by the set of variability indicators. In this way, for instance, we demonstrated that the majority (about 80%) of HRV and arterial pressure variability information, which is distributed across 15–20 variables, can be reduced to only four (“physical”) domains, corresponding to *amplitude* of signal variations (msec or msec^2^), rate of *pulse* (beat/min) and arterial pressure load (mmHg), as well as dimensionless (pure number, as with normalized units) *oscillations.* In the case of adults with obesity, the interested domains are in particular pulse, pressure and amplitude, and not oscillations. Clearly, from these indices it is not possible to obtain measures of autonomic activity, but only indices of autonomic (dys)regulation.

A simplified approach, primarily based on exploratory factor analysis and simple correlations, was employed in the present feasibility study based on a relatively small, but homogeneous, population.

### 2.7. Statistics

Data are presented in the text, figures and tables as averages ± SD.

Statistical evaluation comprised independent *t*-test, simple Pearson’s correlations, and exploratory factor analysis (EFA). Computations were performed with a commercial statistical package (SPSS 27) considering a significance threshold of *p* < 0.05 (two sided).

## 3. Results

Table 1 reports summary anthropometric, hemodynamic and metabolic data in the two groups (normal weight vs. overweight/obesity subjects; i.e., NW vs. OW/OB). There is no difference in height between the two groups, while OW/OB subjects show, by design, a greater weight, BMI and BMI z-score, as well as Waist and Waist/Height, as compared to NW. In addition, as expected, a significant difference between the two groups in almost all metabolic indicators was noted, with the exception of HDL, glucose and TryG.

Resting values of key autonomic indices are presented in Table 2, which shows substantial equivalence of data. Conversely, SAP mean and (borderline) SAP variance are greater in the NW/OB groups.

**Figure 1:**
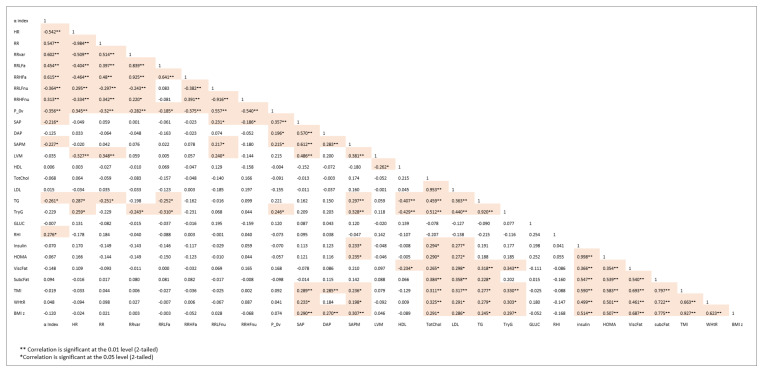
Correlation matrix between major autonomic and metabolic/hemodynamic variables.

Figure 1 reports the correlation matrix between major autonomic and metabolic/hemodynamic variables. Not surprisingly, yet interestingly, there is a clustered significant correlation between homogeneous indices. More specifically, autonomic variables show, as expected, strong intergroup correlations: e.g., alpha index vs. RR variance (r = 0.602), RR LF a (r = 0.454) or vs. RR (r = 0.547). Notably alpha index, a marker of overall gain of cardiac baroreflex, is also significantly correlated with TG (r = −0.261) and endothelial indices (RHI, r = 0.276).

Systolic arterial pressure indices correlate with left ventricular mass, TG (and TryG), insulin and HOMA, TMI, and with WHtR and BMI z-score.

A strong intragroup correlation characterizes clustering of metabolic indicators vs. visceral/subcutaneous fat.

Simple correlations, however, are very sensitive to outliers and cannot delve into the value of congruent correlations. Moreover, collinearity may be a problem. Given the complexity of the “organization of the whole mechanism of control” that we seek to explore, we need to choose “the appropriate technique of investigation” [33], and it is unlikely that simple deterministic models might be appropriate [34]. Accordingly, the bioengineering efforts to provide new “gold standard” indices [35] may have limited clinical relevance.

Conversely, given the practical goals of physiopathological research, it might be useful to consider that a limited number of hidden domains are responsible for the continuous transformation of multiple neurovisceral control functions into the complexity of a human organism [33]. Regarding beat-by-beat peripheral cardiovascular variabilities, prior studies with sympathetic nerve recordings provided evidence of “common central mechanisms governing sympathetic and parasympathetic rhythmic activity” [36]. Therefore, in recent studies we employed modern multivariate statistics to explore the possibility of reducing the large number of individual empirical indices into a smaller set of congruent variables, carrying specific aspects of relevant information. Specifically, in obese adults we observed that EFA suggested four domains (amplitude, frequency, pulse and pressure) [19].

The same approach was employed in the present study.

**Figure 2:**
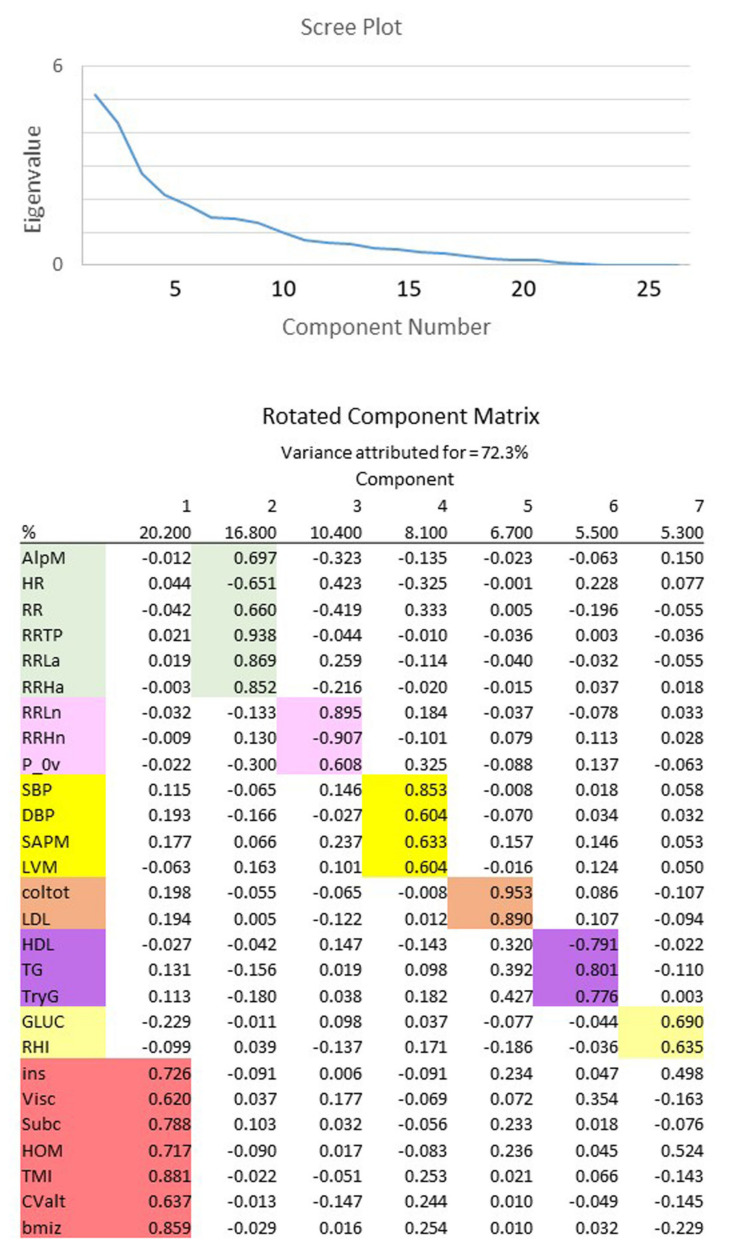
Exploratory factor analysis (EFA) of autonomic and metabolic indices from controls and patients. The seven hidden components that synthetize the reciprocal interrelations between autonomic nervous system regulation and metabolic indicators are reported in a different color.

As shown in Figure 2, applying EFA to multidimensional data allowed us to reduce the number of variables describing the complex relationship between autonomic cardiac regulation and obesity. From an initial set of 27 explicit variables, accounting for 72.3% of data variance, EFA extracts 7 hidden components that more synthetically describe the reciprocal interrelations between ANS regulation and metabolic indicators.

In detail, employing the terminology that we described with this approach (see Methods), factor 1 (about 20% of total variance) links to the *metabolic* domain for the positive correlations with insulin and HOMA, visceral and subcutaneous fat, and TMI, WHtR, and BMI z-score; factor 2 (about 16% of total variance) relates to the *amplitude* and *pulse* domains, because it correlates positively with the alpha index, RR, RR variance, LF absolute and HF absolute, and negatively to HR. Factor 3 (about 10% of total variance) links to purely numerical indices of the *oscillatory* domain (RR LF nu, HF nu and P_0v). Factor 4 (about 8% of total variance) links to the *pressure* domain (SAP, DAP, SAP Mean). The remaining 5, 6 and 7 factors correspond, respectively, to lipids (cholesterol and TG), fasting glucose and endothelium performance. In brief, EFA provides a means to focus on the essential elements of individual factors considering the variable with the highest loading as representative of the information carried by the component. It should be added that for every factor it is possible to observe which empirical variable furnishes the greatest part of information. For instance, in the metabolic cluster (factor 1) the variables with highest loading (TMI and BMI z-score) should be confronted with visceral fat and HOMA that show the lowest loading. This suggests a relatively large variation in the link between empirical data and computed hidden factors. Likewise, for the HRV-derived indices we see factor 2 characterized by amplitude information (such as RR var), while factor 3 provides information on dimensionless (i.e., pure numbers) information. This shows, once more, that normalized variables carry a different meaning from absolute ones [37]. This finding, overall, suggests that autonomic nervous system control represents a pivotal mechanism that is secondary, as is obvious, only to insulin resistance and metabolic indices to describe the effect of obesity in the considered population. The autonomic derangement is more evident on the vascular end.

## 4. Discussion

The rising prevalence of obesity in the young population renders, nowadays, this condition not only a risk factor for many illnesses, particularly diabetes and cardiovascular diseases, but also a social emergency [38]. Accordingly, addressing obesity directly, particularly in young people, has become a must in preventive strategy, where lifestyle changes outperform the usual pharmacological tools considering the young age [39]. As a corollary, advanced, practical knowledge of underlying physiopathological mechanisms, like ANS control, which may be improved by behavioral interventions [14,15,40] such as diet and exercise, becomes mandatory.

This observational study confirms and extends previous observations of ours indicating a generalized involvement of cardiac and arterial systems in youth obesity [41]. In particular, an increase in LV dimensions and mass as well as in common carotid diameter and distension were found, mainly reflecting adaptation to a body-size-induced increase in hemodynamic load, and an increase in carotid intima-media thickness IMT related to both adaptive remodeling and metabolic risk.

Here, we now report a significant correlation between metabolic indices (as triglycerides), endothelial control (as RHI) and autonomic control, particularly in its vascular end (alpha index), which may play a mechanistic role in the above reported changes.

To interpret the alterations of multiple control systems, as in the present study, it is important to recognize that neural visceral regulation is not simply based on purely efferent, antagonistic, sympathetic and parasympathetic innervation (described approximately by a dynamic balance), but beat-by-beat regulation is the result of a complex hierarchical multilevel system [42,43], comprising positive–negative feedback, pointing at a *unitary* functional aim [33,44]. Given this complexity, simple statistics, like correlation, can suggest functional links, but correlation is very sensitive to computational drawbacks, like collinearity. Moreover, it is difficult to provide a clear-cut interpretation of underlying mechanisms. Indeed, even now, HRV derived indices do not have an agreed physiopathological meaning. This applies in particular considering the different way of computing variables (according to a novel systematization of metrics) [37]. According to this view (applicable to other cardiac properties, like ventricular performance), physical indices (having a measurable metric, e.g., heart volume) and numerical quantities (e.g., EF that is dimensionless), obtained with a division (ratio), provide different, possibly complementary, information. In the case of HRV we may consider e.g., RR interval or variance (units are msec or msec^2^) or the normalized LF and HF components, or LF/HF ratio (dimensionless). We reported several years ago that nu variables are more efficient than RR variance in detecting conditions characterized by increased sympathetic activity (as with standing up) [45]. In the present study we report for completeness a table of simple correlations, merely to point out the clustering of congruent variables, and the relatively weak link between variables pertaining to different domains. To make this point, consider the correlation between alpha index (a measure of the cardiac baroreflex) and RHI (a measure of endothelial functionality): the obtained r value of 0.276 might seem small. It is, however, of the same order of magnitude (r = 0.278) as the correlation between arterial pressure and alpha index in humans [46], suggesting that relationships between physiopathological indices may be more complex than those dictated by simple algebra.

A more rewarding way of extracting coded information from cardiovascular variables might be provided by multisystem analysis or multivariate statistics.

We have been exploring this possibility using advanced multifactorial statistical analysis (as exploratory factor analysis, EFA) applied to heart rate variability. We have recently shown that EFA [19,20], while reducing the large number of autonomic indices provided by usual techniques, also extracts a smaller number of hidden variables that carry a functional, integrated, meaning. In the specific case of obesity, in adults, we found that EFA reduces the totality (*n* = 16) of indices obtained from HRV to only five domains, containing more than 80% of the embedded information. According to this approach, the following are the critical elements of autonomic cardiovascular control playing a role in obesity dysautonomia: strong role for *pulse* and *pressure*; almost strong for *alpha index*, weak role for *amplitude* and *oscillations* [19]. It may be added that for these types of data the comparison between groups is better served by considering both differences in position and in distribution. This last aspect may even be prevalent, although it is rather complex to compute, and requires larger study populations.

Although we cannot directly transpose the adult approach to these data from children, we may also confirm that in the present young population pressure and pulse seem to relate strongly to the various metabolic elements of childhood obesity. Moreover, preliminary distribution analysis suggests that, at this young age, metabolism and pressure (which is most sensitive in adults) [19] values are significantly different between normal weight and children with obesity, while cardiac autonomic indices are not (data not presented for simplicity). Thus, we may hypothesize that in childhood obesity the initial stage is characterized by hemodynamic alterations (pressure and endothelial function), but not yet by autonomic cardiac impairment. This hypothesis renders more cogent the introduction of lifestyle changes to reverse the gradual autonomic dysfunction that is present in obese or metabolically unsound individuals [47].

Triglycerides, insulin resistance as per HOMA, and BMI appear linked to arterial pressure indices (Figure 2). Systemic pressure is also, as expected, correlated to LVM. Amplitude and oscillation domains carry a weak relation with metabolic and anthropometric indices of childhood obesity. Conversely, the alpha index shows in children a stronger link with metabolic obesity (TG) and endothelial (RHI) indicators. This analysis might be useful to detect the initial ANS impairment in overweight/obese children, even if unpaired *t*-test analysis, considering the two small groups of this study, fails in identifying significant differences in ANS indices. Indeed, cardiac dysautonomia may take a long time to become manifest [47].

Obesity has a negative, progressive, impact on health, being a risk for many diseases, including cardiovascular disorders. In adults, established obesity is characterized by increased sympathetic activity, with a relative relation with severity of the condition, often associated to arterial hypertension.

Here, we analyzed autonomic cardiovascular control in children with overweight and obesity, showing a substantial equivalence of resting values of key autonomic indices in normal weight and overweight/obesity. Since we also found a clear link between autonomic variables and metabolic indicators, as well as with left ventricle mass, our results support the contention that subtle changes in autonomic nervous system control could be considered a possible way to chase early preclinical cardiovascular derangement in the pediatric obese population, even before the occurrence of frank autonomic dysregulation.

This finding should be interpreted considering that autonomic nervous system imbalance is a potent risk factor for adverse cardiovascular events [11,12,13]. Nevertheless, only a limited number of studies have analyzed the autonomic function in obese pediatric populations [11,12,13,14,48], and an autonomic dysfunction has been reported, as a result of sympathovagal imbalance. In our patients, blood pressure was higher in the overweight/obese group compared to normal weight children, in the absence of substantial differences in autonomic indices. These data suggest the testable hypothesis that a mild increase in blood pressure may be considered an early sign of cardiovascular dysregulation, expressing early derangement of vascular health (as suggested by the altered RHI). The occurrence of endothelial dysfunction before the appearance of structural arterial damage and autonomic impairment fits well with Joyner’s [40] “risk factor gap”, as a strong support for exercise and diet as early preventive treatments. Obesity-related hypertension is associated with increased cardiovascular morbidity and mortality, and early diagnosis and treatment for blood pressure control and weight reduction is essential [49].

Hypertension often coexists with IR [50]. IR may contribute to increased blood pressure through several mechanisms, including the enhanced tissue angiotensin II and aldosterone activities, increased sympathetic nervous system activity and oxidative stress; the genetic predisposition for IR and hypertension could be also considered [51]. The links between autonomic indices and insulin levels and HOMA-IR could confirm the role of hyperinsulinemia in the sympathetic and vagal activity leading to hypertension [52]. In our young population we may only witness the initial derangement of cardiovascular regulation, which may progress from arterial dysregulation to increased arterial pressure, and subsequently comprise altered cardiac autonomic regulation. In our study, the link between visceral and subcutaneous fat and adiposity indices and major autonomic variables confirmed that obesity itself leads to cardiovascular alterations [19]. Adipose tissue is an endocrine organ modulating an inflammatory state and metabolic processes leading to consequences of the cardiovascular system [53] that we have not addressed here.

ANS impairment characterizes many chronic diseases in adulthood, such as diabetes, arterial hypertension and coronary artery disease [30,46], being one of the most important targets of preventive strategies [39,54,55]. Overweight and obesity in children, although negative as a clinical risk factor, represent, however, conditions that are easy to manage by employing behavioral lifestyle interventions. These strategies, further to other metabolic, immunological and hemodynamic benefits, are capable of improving autonomic nervous system impairment [14,15,40].

A few study limitations should be acknowledged, starting from the relatively small sample size that can limit power analysis. Therefore, further large population studies are mandatory. Additionally, this is a retrospective study and results may be affected by some bias. Finally, no family history on cardiovascular diseases and/or other potential cardiovascular risk factors, such as nutritional behavior, physical activity level and prenatal environment were analyzed.

## 5. Conclusions

Pediatric patients with obesity show a link between indices of autonomic and endothelial function, fat distribution and metabolic profile. A greater blood pressure value at rest in control compared to overweight/obese groups was detected in the presence of substantial equivalence of resting values of autonomic indices. Even though the strategies to reduce cardiovascular risk have not yet been fully elucidated, the role of autonomic control optimization to prevent, or at least delay, further deterioration and the occurrence of structural vascular damage, should be not excluded. This multidimensional analysis might help to better characterize the individual progression of the various aspects of altered metabolic, hemodynamic and cardiac autonomic regulation.

## Figures and Tables

**Table 1:** Anthropometric, hemodynamic and metabolic data in normal weight (NW) and overweight/obese children (OW/OB).

Variables	NW	OW/OB	Significance
Height (cm)	158.9 ± 12.2	157.6 ± 11.6	0.549
Weight (kg)	48.9 ± 11.3	72.3 ± 18.7	<0.001
BMI (kg/m^2^)	19.07 ± 2.04	28.8 ± 5.51	<0.001
BMI z score (-)	0.11 ± 0.65	2.75 ± 1.09	<0.001
Triponderal Mass Index (kg/m^3^)	12.01 ± 0.95	18.34 ± 3.56	<0.001
Waist circumference (cm)	72.0 ± 10.1	94.2 ± 14.9	<0.001
WHtR (-)	0.41 ± 0.14	0.59 ± 0.11	<0.001
Visceral fat (cm)	2.14 ± 0.71	4.08 ± 1.31	<0.001
Subcutaneous fat (cm)	1.03 ± 0.51	3.49 ± 0.98	<0.001
SAP (mmHg)	107.4 ± 11.6	113.7 ± 10.2	0.003
DAP (mmHg)	63.8 ± 6.6	66.5 ± 6.3	0.032
SAP (percentile)	43.9 ± 26.9	62.6 ± 29.2	<0.001
DAP (percentile)	48.5 ± 19.3	58.2 ± 17.3	0.006
HR (b/min)	71.8 ± 9.5	70.0 ± 9.9	0.324
LV mass (g)	119.4 ± 43.0	122.9 ± 36.0	0.716
RHI (-)	1.86 ± 0.48	1.59 ± 0.44	0.037
Total cholesterol (mg/dL)	130.5 ± 39.7	165.8 ± 35.9	0.001
LDL cholesterol (mg/dL)	73.4 ± 32.8	100.4 ± 30.8	0.003
HDL cholesterol (mg/dL)	46.8 ± 12.5	47.3 ± 12.2	0.868
Triglycerides (mg/dL)	51.5 ± 20.0	89.8 ± 68.0	0.030
TryGI (mg/dL)^2^	7.62 ± 0.44	7.96 ± 0.58	0.145
Fasting glucose (mg/dL)	78.5 ± 7.7	79.0 ± 11.0	0.935
Insulin (pmol/l)	48.0 ± 18.4	128.7 ± 83.6	<0.001
HbA1c (mmol/mol)	5.16 ± 0.28	5.43 ± 0.31	0.001
HOMA-iR	0.89 ± 0.31	2.25 ± 1.41	<0.001

Data are presented as means ± SD. Abbreviations: BMI = body mass index; WHtR = waist/height ratio; SAP = systolic arterial pressure, DAP = diastolic arterial pressure, HR = heart rate, LV = left ventricle; RHI = reactive hyperemia index; LDL = low density lipoproteins; HDL = high density lipoproteins; TryGI = triglyceride-glucose index; HbA1c = glycated haemoglobin; HOMA-IR = homeostatic model assessment for insulin resistance.

**Table 2:** Resting values of key autonomic indices from RR interval variability in normal weight (NW) and overweight/obese children (OW/OB).

Variables	NW	OW/OB	Significance
Alpha index (ms/mmHg)	28.0 ± 19.0	24.2 ± 13.2	0.203
RR interval (ms)	849.9 ± 118.8	874.2 ± 125.8	0.304
RR variance (ms^2^)	4806.2 ± 5381.2	5894.5 ± 6311.5	0.340
RR LF a (ms^2^/Hz)	1199.1 ± 1372.7	2423.9 ± 3635.9	0.166
RR HF a (ms^2^/Hz)	2365.3 ± 3824.9	2423.9 ± 3635.9	0.934
RR LF nu (-)	39.8 ± 20.4	42.0 ± 17.9	0.534
RR HF nu (-)	53.0 ± 21.1	48.6 ± 18.3	0.235
RR LF/HF (-)	1.27 ± 1.75	1.28 ± 1.52	0.976
RR LF f (Hz)	0.10 ± 0.03	0.10 ± 0.02	0.374
RR HF f (Hz)	0.30 ± 0.07	0.32 ± 0.06	0.113
*p*_0v (-)	15.6 ± 9.9	17.4 ± 12.6	0.409
Resp HF f (Hz)	0.31 ± 0.06	0.33 ± 0.06	0.139
SAP Mean (mmHg)	106.1 ± 12.3	113.6 ±11.1	0.001
SAP total power (mmHg^2^)	24.2 ± 18.9	32.1 ± 25.2	0.059

Abbreviations; RR = interval between successive R waves; LF = low frequency; a = raw value; HF = high frequency; f = frequency; Hz = Hertz; *p* = beat sequence; Resp = respiration; SAP = systolic arterial pressure.

## Data Availability

The data presented in this study are available on request from the corresponding author.
